# Central New York Homœopathic Medical Society

**Published:** 1883-06

**Authors:** 


### CENTRAL NEW YORK HOMŒOPATHIC MEDICAL SOCIETY.

A quarterly meeting of the Central New York Homœopathic Medical Society was held at Syracuse, N. Y., March 15th, 1883, Dr. C. W. Boyce, of Auburn, N. Y., in the chair.

Dr. D. W. Clausen, of Auburn, N. Y., delivered the following address, and read the report of the Committee appointed at last meeting to draw up a protest against "that resolution" of the American Institute touching "freedom of medical opinion and action."

### Dr. D. W. Clausen's Address.

Mr. President and Gentlemen: From the early dawn of civilization, as science and art have kept pace with the onward march of culture and refinement, sagacious men and women, moved by lofty aspirations, have at various times formed themselves into societies or bodies, respectively with their several scientific and artistic pursuits. These associations have been designed not only for mutual benefit, but also that the seeds of knowledge might be disseminated among others of the world, with a view to more extended usefulness as well as to development and progress.

Apart from the spiritual welfare of mankind, nothing is of so much concern regarding the interests of the world at large as is that which pertains to health and longevity, with the happiness these are able to afford. Accordingly, the ministers of human health as far back, I believe, as when the ministry of health was almost exclusively the privilege of a priesthood, have been uniting themselves into bodies for the purpose of developing knowledge in that sphere which so momentously concerns the welfare of suffering humanity. And this indeed is wise, for the words of our own Hahnemann force admission within our ears: "In a science in which the welfare of mankind is concerned any neglect to make ourselves masters of it is a crime."

Now, with regard to the objects of usefulness, development, and progress in the formation of societies, it is evident that, for these to be successfully accomplished, some essential tenets must be the rule of doctrine and the unerring guide to development.

These tenets, therefore, naturally involve three propositions:

1st. That which is called a science can be a science only as it is governed by *Law*.

2d. Law, *to be Law*, must be *universally* applicable to the science it governs.

3d. Any deviation from that *Law* is unscientific and empirical, and can reap only spurious results.

Gentlemen, we, as a society, are to do with the science and art of *medicine*, and we desire to-day to see whether all that has been accomplished by medical associations, and especially by those denominated *Homœopathic*, has been according to the essential tenets of the healing art; whether the tendency has been retrogressive or toward development. Let us briefly scan the history, the proceedings, and the tendency of the oldest national medical association in this country—The American Institute of Homœopathy.

This association was organized when, as Carroll Dunham eloquently says, "the step of civilization yet hesitated at the eastern verge of the prairies." Its founders were noble men—men full of truth—men of moral courage sufficient to lift them above the derision of old-school scoffers, not caring for the "Recognition." Among the articles of its constitution we find the object of the Institute to be "the promotion of medical science." Its founders recognized for that science a *Law;* they recognized that Law to be "*Similia Similibus Curantur;*" they therefore logically recognized Homœopathy as "The science of therapeutics, being a *science* by virtue of having a *Law.*"

It therefore follows from this that "the promotion of medical science" can result only from strict conformity to the *Law.*

'Twas on a fine summer's day at Chicago, when as yet but five months had passed in the memorable year of 1870, that our much beloved and lamented Carroll Dunham, as President of the American Institute, delivered his annual address—an address that has been no less unfortunate than eloquent: Unfortunate, because of the dire results to be hereafter shown; eloquent, because of the grand principle and truth which ring so sweetly therein, like the song of Heaven in the ears of the faithful. In that address our Dunham said: "Mr. President and my colleagues, my own position on these points of doctrine is not unknown to some of you. Holding that the law, *Similia Similibus Curantur*, expresses the relation between the specific drug action and the diseased organism, and that it is a sufficient and the only trustworthy guide in every application of drugs to cure the sick, I fully believe not only that the practical rules laid down by Hahnemann, which enjoin the single remedy and the minimum dose, are the rules of sound practice, but I believe that every *observing physician who faithfully applies the law of cure** will be led by experience to the same conclusion, and will adopt these rules as leading to the best results. Notwithstanding this belief, I advocate entire liberty of opinion and practice. Nay, *because of this belief*, I plead for liberty, for I am sure perfect liberty will the sooner bring knowledge of the truth and that purity of practice which we all desire."

What grand words! It is only the *perfect law* of liberty that delivers us from the bondage of lawlessness and recklessness with their direful punishments. The lawless are always slaves to their own waywardness; they enjoy *license*, but not the perfect law of liberty. Carroll Dunham proved, not only by his words, but also by his faithful practice, that he loved the liberty of *Law*, and that he hated all

---

* Quotations not italicized in the original have been so emphasized in this address and protest for an obvious reason. D. W. C.

disobedience to law. Hear another part of his eloquent speech: "I thank God for a band, by no means contemptible in numbers, of the *strict followers of Hahnemann.*" Again, said he, "There are among those who call themselves homœopathists some who are impostors; men unworthy to be called physicians; men without knowledge and without conscience, who play upon the credulity of mankind, and pervert to their own aggrandizement every trust committed to them. That such men, professing to belong to our school, should be regarded by the community as belonging to it, is certainly a misfortune."

Mr. President and gentlemen, I need hardly speak of the willful misinterpretation of that noble President's words, and the perversion of their truth, in his plea for liberty. All of you are aware what strenuous efforts have been made by certain giddy-headed communists within our ranks to unite the common school of medicine with ours. We have deplored the inconsistency and the irrationality of this crazy idea. We have read—O shade of Hahnemann!—we have read that disgraceful and abominable resolution which was "passed" at the last meeting of the Institute, in 1882—"passed," it would seem, as if only by reason of a mean advantage taken to introduce it when, at the "tail-end" of the meeting, but very few members were left to decide as to the wisdom of so stupendous a suggestion! This is the grand climax of the perversion of Carroll Dunham's plea for liberty. They have unfurled the flag at the masthead of their disloyalty; it is the flag of treason; on one side is stamped "Liberty;" on the other side, "Recognition." These men delight in old-school recognition, but they cannot be consistently loved by the *honest*, even in the common school of medicine.

Mr. President and gentlemen, the company of us here gathered represents the "Central New York Homœopathic Medical Society." There are some of you present who are probably more acquainted with the object of this Society than you are with its history; and it may stimulate you with encouragement and for present needed action, to give you a cursory glance at its origin and history, which are long in the memory of some who honor us with their presence to-day. I copy from the *Transactions of the World's Homœopathic Convention*, of 1876, the following:

"A number o. homœopathic physicians of Central New York met in Syracuse, September 13th, 1849, for the purpose of promoting the interests of Homœopathy. Dr. A. L. Kellogg, of Bridgewater, was called to the chair. A committee was appointed to correspond with the homœopathic physicians of Central New York, and to call a meeting to perfect a plan of organization. In response to the call, a number of these convened at the National Hotel, in Utica, N. Y., January 16th, 1850. The Convention then proceeded to organize a 'Homœopathic Medical Society of Central New York,' auxiliary to the American Institute of Homœopathy: A Constitution and By-laws were adopted, and twenty-nine names were signed thereto." Our honored and venerable Dr. L. B. Wells, who is with us to-day, was one of its first officers—vice-president. "The undersigned members of that early organization avowed their firm belief, as follows:

"1st. In the universality of the therapeutic law—*Similia Similibus Curantur.*

"2d. In the superior efficacy and safety of *pure homœopathic practice*, in contradistinction to every other system or combination of systems.

"3d. In the great therapeutic value and power of those preparations denominated *potentized medicines.*"

From a variety of causes, however, this organization died away.

The first Tuesday in May, 1866, after the adjournment of the Onondaga County Medical Society, several physicians from different parts of Central New York held a meeting for the purpose of organizing a society. The inception of it was in this wise: Drs. T. D. Stowe, A. B. Morgan, and (the one who now graces our chair), Dr. C. W. Boyce, while returning from the annual meeting of the State Society, in 1866, thought it would be pleasant and beneficial for the physicians of Central New York to come together for mutual improvement. A preliminary meeting was formed, and by a rather striking coincidence, our present Chairman was then called to the chair. This, fellow-members, is, in brief, the history of the Society to which we have the honor to belong. The *honor*, I say, because the testimony stands yet ineffaced and ineffaceable from record, that "Through all vicissitudes this Society has adhered firmly to the fundamental principles of Homœopathy, viz.: The *Simillimum*, the *single remedy*, and the *Minimum dose.*"

Mr. President and members of "The Central New York Homœopathic Medical Society"—the *Society representative* of pure Homœopathy in America, if not in the whole world—is not this admirable standing of our association, as shown in the brief sketch given, sufficient to awaken us to the "great responsibility" involved by our liberty?—does it not impose on us the solemn duty to protest with emphasis against every departure from ever-efficient Homœopathy and its unerring law, which we have cherished for these many years? I say unto you, that in honor to Truth and Loyalty, it is verily our duty.

With regard to that most absurd of all steps yet taken by the Institute, in the adoption of that resolution passed at its last session, touching "Absolute freedom of medical opinion and unrestricted liberty of action," a suggestion was offered at our last meeting as to the propriety of this Society protesting against this resolution so detrimental to the interests of Homœopathy; and it was your good pleasure to appoint a Committee for the purpose of reporting on the subject. Your Committee therefore beg leave now to say that they have with diligent care considered the nature of the said resolution, and herewith respectfully submit the following preamble and resolution, which, in their judgment, may best serve to express the sentiments of this Society.

D. W. Clausen.

### PREAMBLE AND RESOLUTION.

Whereas, *Obedience to law* is the only liberty possible in the nature of things, more than that being only license; and,

Whereas, The use of drugs for the cure of the sick can be successful only when they are administered in obedience to law; and,

Whereas, We believe *that* law to be "Similia Similibus Curantur," the sole foundation of Homœopathy; and,

Whereas, The American Institute of Homœopathy at its last meeting passed the following resolution, to wit:

"Resolved, that it is the sense of the American Institute of Homœopathy that no physician can properly sustain the responsibilities or fulfill all the duties of his professional relations, unless he enjoys absolute freedom of medical opinion and unrestricted liberty of action, as provided in the code of ethics of this Institute;" and

Whereas, The said resolution, in our opinion, gives unbounded license to all sorts of disobedience to the law of cure; and,

Whereas, The said American Institute of Homœopathy is set for a pure materia medica and a knowledge of Homœopathy; therefore,

*Resolved,* That we, The Central New York Homœopathic Medical Society, greatly deplore the adoption of the said resolution, as we believe it to be contrary to the principles of the said "Institute" and wholly subversive of the very object of its existence; and that we respectfully and most earnestly urge the said American Institute of Homœopathy at its earliest opportunity to rescind the said resolution and expunge it from its records.

D. W. CLAUSEN,<br>W. A. HAWLEY,<br>E. P. HUSSEY, } *Committee on Protest.*

The Society adopted the preamble and resolution, and accepted, with thanks, the address of Dr. Clausen.

C. P. JENNINGS, *Secretary.*

